# Redetermination of (η^4^-*s-cis*-1,3-butadiene)tricarbonyl­iron(0)

**DOI:** 10.1107/S1600536810039218

**Published:** 2010-10-09

**Authors:** Guido J. Reiss

**Affiliations:** a Institut für Anorganische Chemie und Strukturchemie, Lehrstuhl II: Material- und Strukturforschung, Heinrich-Heine-Universität Düsseldorf, Universitätsstrasse 1, D-40225 Düsseldorf, Germany

## Abstract

The crystal structure of the title compound, [Fe(C_4_H_6_)(CO)_3_], was previously reported by Mills & Robinson [*Acta Cryst.* (1963) ▶, **16**, 758–761]. The compound crystallizes in the centrosymmetric space goup *Pnma* with the complex located on a mirror plane. The redetermination of this structure at 100 K yielded almost equilibrated C—C bond lengths within the butadiene ligand according to a metal-to-ligand bonding–back-bonding mechanism. The C—C bond lengths presented herein are significantly shorter than those reported earlier. The H-atom positions that have not been reported so far were located by difference Fourier maps. The positional parameters of all H atoms and individual *U*_iso_ values were refined freely.

## Related literature

For {Fe(CO)_3_} compounds and applications, see: Knölker (2000 ▶); Pearson (1983 ▶); Sawyer *et al.* (2008 ▶ and references therein). For theoretical and experimental data for η^4^-*s*-*cis*-1,3-butadienetricarbonyl­iron(0), see: Bühl & Thiel (1997 ▶); Reihlen *et al.* (1930 ▶); Mills & Robinson (1963 ▶); Kukolich *et al.* (1993 ▶); Kruczynski & Takats (1976 ▶). For related complexes, see: Reiss & Konietzny (2002 ▶); Davidson (1969 ▶); Immirzi & Allegra (1969 ▶); Porri *et al.* (1965 ▶); Reiss (2002 ▶). For librational corrected values for C—C bond lengths, see: Schomaker & Trueblood (1968 ▶).
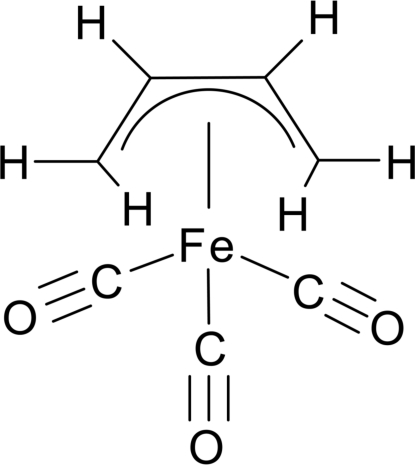


## Experimental

### 

#### Crystal data

[Fe(C_4_H_6_)(CO)_3_]
*M* *_r_* = 193.97
Orthorhombic, 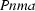

*a* = 11.4323 (6) Å
*b* = 10.9146 (6) Å
*c* = 6.1664 (4) Å
*V* = 769.44 (8) Å^3^
*Z* = 4
Mo *K*α radiation
μ = 1.91 mm^−1^
*T* = 100 K
0.40 × 0.38 × 0.36 mm

#### Data collection

Oxford Diffraction Xcalibur Eos diffractometer
Absorption correction: multi-scan (*CrysAlis PRO*; Oxford Diffraction, 2009 ▶) *T*_min_ = 0.850, *T*_max_ = 1.000
13021 measured reflections
1177 independent reflections
1131 reflections with *I* > 2σ(*I*)
*R*_int_ = 0.019
3 reference frames every 30 min  intensity decay: none

#### Refinement

*R*[*F* ^2^ > 2σ(*F* ^2^)] = 0.015
*wR*(*F* ^2^) = 0.040
*S* = 1.04
1177 reflections
67 parameters
All H-atom parameters refined
Δρ_max_ = 0.35 e Å^−3^
Δρ_min_ = −0.22 e Å^−3^

### 

Data collection: *CrysAlis PRO* (Oxford Diffraction, 2009 ▶); cell refinement: *CrysAlis PRO*; data reduction: *CrysAlis PRO*; program(s) used to solve structure: *SHELXS97* (Sheldrick, 2008 ▶); program(s) used to refine structure: *SHELXL97* (Sheldrick, 2008 ▶); molecular graphics: *DIAMOND* (Brandenburg, 2010 ▶); software used to prepare material for publication: *publCIF* (Westrip, 2010 ▶).

## Supplementary Material

Crystal structure: contains datablocks I, global. DOI: 10.1107/S1600536810039218/nc2198sup1.cif

Structure factors: contains datablocks I. DOI: 10.1107/S1600536810039218/nc2198Isup2.hkl

Additional supplementary materials:  crystallographic information; 3D view; checkCIF report

## Figures and Tables

**Fig. 1.**
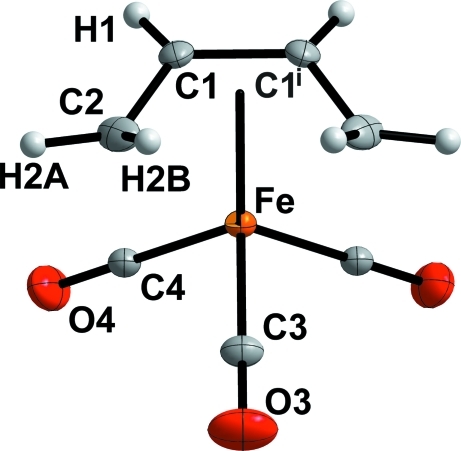
Hydrogen atoms are drawn with an arbitrary radius and the displacement ellipsoids are shown at the 50% probability level. Symmetrie codes: i = x, -y+1/2, z.

## supplementary crystallographic information

### Comment

Olefine complexes containing the {Fe(CO)_3_} fragment are intensively studied and are used as catalysts in a wide range of applications (Knöker, 2000; Sawyer *et al.*, 2008). Diene and dienyl complexes of iron are common intermediates in iron catalyzed organic reactions (Pearson, 1981). Butadiene complexes have been structurally characterized since the 60 s of the last century (Porri, Lionelli, Allegra & Immirzi, 1965; Immirzi & Allegra, 1969). The first synthesis of the title compound dates back to the 30 s of the last century (Reihlen *et al.* 1930). Vibrational spectroscopic (Davidson, 1969) and ^13^C-NMR spectroscopic studies (Kruczynski & Takats, 1976) were undertaken on the title compound. The structures of the title compound were derived from microwave spectroscopy (Kukolich *et al.*, 1993) and quantum chemical calculations (Bühl und Thiel, 1997). The first crystal structure verified the constitution of the title complex consisting of three coordinated CO ligands and one η^4^-coordinated butadiene ligand (Mills & Robinson, 1963). This early structure determination, based on equi-inclination photographic data, did not report any information on the hydrogen atom positions. The standard uncertainties of the reported C—C bond lengths that range from 0.05 to 0.06 do not allow a detailed discussion of the bonding properties of the butadiene ligand.

The redetermination of η^4^-*s*-*cis*-1,3-butadienetricarbonyliron(0) at 100 K yielded significantly improved geometric parameters that enables a detailed discussion of the structure. The Fe(0) center of the title complex is coordinated by three carbonyl ligands and one *s-cis*-1,3-butadiene ligand. The different *C*—Fe(carbonyl) and C≡O bond lengths are equal within their standard uncertainties. The angles between the C≡O ligands are 93.11 (6) and 101.50 (4)°. The coordinated 1,3-butadiene ligand shows the well known *s-cis-*conformation with the C—C bond lengths equilibrated according to the s.u.'s derived from the X-ray diffraction experiment. The C—C bond lengths are significant shorter (C1—C2 1.423 (1); C1—C1^i^ 1.418 (2) (librational corrected values; Schomaker and Trueblood, 1968)) than those reported for the crystal structure of this compound but they are in very good agreement to values derived from microwave spectra (Kukolich *et al.*, 1993) and theoretical calculations (Bühl & Thiel, 1997). The values are in good agreement to values derived for the analogous bis(1,3-cyclohexadiene)monocarbonyliron(0) complex (Reiß, 2002). This equilibration is a consequence of a bonding-back bonding mechanism between the diene ligand and the metal center (Reiß & Konietzny, 2002).

The coordination figure at Fe(0) is best described as a square pyramid with the sterically more demanding *s-cis*-1,3-butadiene ligand occupying two coordination sites of the basis. The hydrogen atom of the coordinated *s-cis*-1,3-butadiene ligand are diplaced of the plane defined by its four carbon atoms. H1 and H2a are only slightly displaced by 11.8 (5)° and 11.1 (6)°, respectively, whereas H2b shows a dihedral angle of 43.4 (7)°. The refined hydrogen atom positions are in very good agreement to results from microwave spectroscopy (Kukolich *et al.*, 1993).

This redetermination at low temperature yielded improved and significant shorter C—C bond lengths and locates the not yet reported hydrogen atom positions for the *s-cis-*1,3-butadiene ligand by crystallographic methods. All geometric parameters derived are in very good agreement with other experimental and theoretical results reported in the last decades.

### Experimental

The title compound was synthesizes according to procedures reported in the literature (Reihlen *et al.*, 1930). The low melting Fe(0) complex crystallizes readily as block shaped crystals on slow cooling.

### Refinement

All hydrogen atoms were located from difference Fourier synthesis. In the final refinement the positional parameters of all atoms, the anisotropic displacement parameters of all non-hydrogen atoms and the *U*_iso_ values of all hydrogen atoms were refined freely.

### Crystal data

**Table:**

[Fe(C_4_H_6_)(CO)_3_]	*F*(000) = 392
*M**_r_* = 193.97	*D*_x_ = 1.674 Mg m^−^^3^
Orthorhombic, *P**n**m**a*	Mo *K*α radiation, λ = 0.71073 Å
Hall symbol: -P 2ac 2n	Cell parameters from 10152 reflections
*a* = 11.4323 (6) Å	θ = 3.3–31.1°
*b* = 10.9146 (6) Å	µ = 1.91 mm^−^^1^
*c* = 6.1664 (4) Å	*T* = 100 K
*V* = 769.44 (8) Å^3^	Prism, yellow
*Z* = 4	0.40 × 0.38 × 0.36 mm

### Data collection

**Table:**

Oxford Diffraction Xcalibur Eos diffractometer	1131 reflections with *I* > 2σ(*I*)
Radiation source: fine-focus sealed tube	*R*_int_ = 0.019
graphite	θ_max_ = 30.0°, θ_min_ = 3.6°
ω scans	*h* = −16→16
Absorption correction: multi-scan (*CrysAlis PRO*; Oxford Diffraction, 2009)	*k* = −15→15
*T*_min_ = 0.850, *T*_max_ = 1.000	*l* = −8→8
13021 measured reflections	3 standard reflections every 30 min
1177 independent reflections	intensity decay: none

### Refinement

**Table:**

Refinement on *F*^2^	Primary atom site location: structure-invariant direct methods
Least-squares matrix: full	Secondary atom site location: difference Fourier map
*R*[*F*^2^ > 2σ(*F*^2^)] = 0.015	Hydrogen site location: difference Fourier map
*wR*(*F*^2^) = 0.040	All H-atom parameters refined
*S* = 1.04	*w* = 1/[σ^2^(*F*_o_^2^) + (0.02*P*)^2^ + 0.3*P*] where *P* = (*F*_o_^2^ + 2*F*_c_^2^)/3
1177 reflections	(Δ/σ)_max_ = 0.001
67 parameters	Δρ_max_ = 0.35 e Å^−^^3^
0 restraints	Δρ_min_ = −0.22 e Å^−^^3^

### Special details

**Table:**

Experimental. Absorption correction details: CrysAlisPro, Oxford Diffraction Ltd., Version 1.171.33.52 Empirical absorption correction using spherical harmonics, implemented in SCALE3 ABSPACK scaling algorithm.
Geometry. All e.s.d.'s (except the e.s.d. in the dihedral angle between two l.s. planes) are estimated using the full covariance matrix. The cell e.s.d.'s are taken into account individually in the estimation of e.s.d.'s in distances, angles and torsion angles; correlations between e.s.d.'s in cell parameters are only used when they are defined by crystal symmetry. An approximate (isotropic) treatment of cell e.s.d.'s is used for estimating e.s.d.'s involving l.s. planes.
Refinement. Refinement of *F*^2^ against ALL reflections. The weighted *R*-factor *wR* and goodness of fit *S* are based on *F*^2^, conventional *R*-factors *R* are based on *F*, with *F* set to zero for negative *F*^2^. The threshold expression of *F*^2^ > σ(*F*^2^) is used only for calculating *R*-factors(gt) *etc*. and is not relevant to the choice of reflections for refinement. *R*-factors based on *F*^2^ are statistically about twice as large as those based on *F*, and *R*- factors based on ALL data will be even larger.

### Fractional atomic coordinates and isotropic or equivalent isotropic displacement parameters (Å^2^)

**Table:**

	*x*	*y*	*z*	*U*_iso_*/*U*_eq_	
Fe	0.422257 (14)	0.2500	0.10001 (3)	0.01195 (6)	
C1	0.40416 (9)	0.31478 (9)	0.41329 (14)	0.02025 (18)	
H1	0.3372 (11)	0.3551 (12)	0.451 (2)	0.025 (3)*	
C2	0.50116 (10)	0.37495 (11)	0.31685 (16)	0.0293 (2)	
H2A	0.4931 (13)	0.4628 (14)	0.294 (2)	0.039 (4)*	
H2B	0.5783 (12)	0.3456 (14)	0.346 (2)	0.033 (4)*	
C3	0.54866 (12)	0.2500	−0.0711 (2)	0.0220 (2)	
O3	0.63206 (10)	0.2500	−0.1730 (2)	0.0381 (3)	
C4	0.33599 (8)	0.36948 (8)	−0.02057 (14)	0.01764 (16)	
O4	0.28356 (7)	0.44729 (7)	−0.09743 (12)	0.02909 (17)	

### Atomic displacement parameters (Å^2^)

**Table:**

	*U*^11^	*U*^22^	*U*^33^	*U*^12^	*U*^13^	*U*^23^
Fe	0.01180 (8)	0.01336 (9)	0.01068 (9)	0.000	0.00004 (5)	0.000
C1	0.0273 (4)	0.0212 (4)	0.0123 (4)	−0.0003 (3)	−0.0005 (3)	−0.0032 (3)
C2	0.0371 (5)	0.0323 (5)	0.0185 (4)	−0.0166 (4)	−0.0030 (4)	−0.0046 (4)
C3	0.0174 (5)	0.0313 (7)	0.0174 (5)	0.000	−0.0004 (4)	0.000
O3	0.0198 (5)	0.0651 (8)	0.0294 (6)	0.000	0.0089 (4)	0.000
C4	0.0198 (4)	0.0180 (4)	0.0151 (4)	0.0000 (3)	0.0025 (3)	0.0001 (3)
O4	0.0338 (4)	0.0254 (4)	0.0280 (4)	0.0108 (3)	0.0010 (3)	0.0072 (3)

### Geometric parameters (Å, °)

**Table:**

Fe—C4^i^	1.7961 (9)	C1—C2	1.4194 (14)
Fe—C4	1.7961 (9)	C1—C1^i^	1.4142 (19)
Fe—C3	1.7893 (14)	C1—H1	0.912 (13)
Fe—C1	2.0675 (9)	C2—H2A	0.974 (15)
Fe—C1^i^	2.0675 (9)	C2—H2B	0.955 (14)
Fe—C2	2.1123 (10)	C3—O3	1.1418 (18)
Fe—C2^i^	2.1123 (10)	C4—O4	1.1425 (11)
			
C4^i^—Fe—C4	93.11 (6)	C1—Fe—C2^i^	70.86 (4)
C4^i^—Fe—C3	101.50 (4)	C1^i^—Fe—C2^i^	39.69 (4)
C4—Fe—C3	101.50 (4)	C2—Fe—C2^i^	80.43 (7)
C4^i^—Fe—C1	125.46 (4)	C2—C1—C1^i^	117.56 (6)
C4—Fe—C1	94.79 (4)	C2—C1—Fe	71.86 (5)
C3—Fe—C1	129.18 (5)	C1^i^—C1—Fe	70.00 (3)
C4^i^—Fe—C1^i^	94.79 (4)	C2—C1—H1	122.6 (8)
C4—Fe—C1^i^	125.46 (4)	C1^i^—C1—H1	118.8 (8)
C3—Fe—C1^i^	129.18 (5)	Fe—C1—H1	119.0 (8)
C1—Fe—C1^i^	40.00 (5)	C1—C2—Fe	68.46 (5)
C4^i^—Fe—C2	164.87 (4)	C1—C2—H2A	116.3 (9)
C4—Fe—C2	91.59 (4)	Fe—C2—H2A	120.3 (9)
C3—Fe—C2	91.63 (4)	C1—C2—H2B	119.2 (9)
C1—Fe—C2	39.69 (4)	Fe—C2—H2B	107.2 (9)
C1^i^—Fe—C2	70.86 (4)	H2A—C2—H2B	116.4 (13)
C4^i^—Fe—C2^i^	91.59 (4)	O3—C3—Fe	177.25 (13)
C4—Fe—C2^i^	164.87 (4)	O4—C4—Fe	178.28 (8)
C3—Fe—C2^i^	91.63 (4)		

Symmetry codes: (i) *x*, −*y*+1/2, *z*.
